# Identifying miRNA-mRNA regulatory networks on extreme n-6/n-3 polyunsaturated fatty acid ratio expression profiles in porcine skeletal muscle

**DOI:** 10.1371/journal.pone.0283231

**Published:** 2023-05-04

**Authors:** Yron Joseph Yabut Manaig, Lourdes Criado-Mesas, Anna Esteve-Codina, Emilio Mármol-Sánchez, Anna Castelló, Armand Sánchez, Josep M. Folch

**Affiliations:** 1 Departament de Ciència Animal i dels Aliments, Universitat Autònoma de Barcelona, Bellaterra, Barcelona, Spain; 2 Plant and Animal Genomics, Centre for Research in Agricultural Genomics (CRAG), CSIC-IRTA-UAB-UB Consortium, Bellaterra, Barcelona, Spain; 3 Department of Veterinary Medicine and Animal Sciences, Università degli Studi di Milano, Lodi, Italy; 4 CNAG-CRG, Centre for Genomic Regulation (CRG), Barcelona Institute of Science and Technology (BIST), Barcelona, Spain; 5 Department of Molecular Biosciences, Science for Life Laboratory, The Wenner-Gren Institute, Stockholm University, Stockholm, Sweden; 6 Centre for Palaeogenetics, Stockholm, Sweden; University of Minnesota Medical School, UNITED STATES

## Abstract

Omega-3 (n-3) and omega-6 (n-6) polyunsaturated fatty acids (PUFAs) are essential fatty acids with antagonistic inflammatory functions that play vital roles in metabolic health and immune response. Current commercial swine diets tend to over-supplement with n-6 PUFAs, which may increase the likelihood of developing inflammatory diseases and affect the overall well-being of the animals. However, it is still poorly understood how n-6/n-3 PUFA ratios affect the porcine transcriptome expression and how messenger RNAs (mRNAs) and microRNAs (miRNAs) might regulate biological processes related to PUFA metabolism. On account of this, we selected a total of 20 Iberian × Duroc crossbred pigs with extreme values for n-6/n-3 FA ratio (10 high vs 10 low), and *longissimus dorsi* muscle samples were used to identify differentially expressed mRNAs and miRNAs. The observed differentially expressed mRNAs were associated to biological pathways related to muscle growth and immunomodulation, while the differentially expressed microRNAs (*ssc-miR-30a-3p*, *ssc-miR-30e-3p*, *ssc-miR-15b* and *ssc-miR-7142-3p*) were correlated to adipogenesis and immunity. Relevant miRNA-to-mRNA regulatory networks were also predicted (i.e., *mir15b* to *ARRDC3*; *mir-7142-3p* to *METTL21C*), and linked to lipolysis, obesity, myogenesis, and protein degradation. The n-6/n-3 PUFA ratio differences in pig skeletal muscle revealed genes, miRNAs and enriched pathways involved in lipid metabolism, cell proliferation and inflammation.

## Introduction

Dietary concentration of polyunsaturated fatty acids (PUFAs) can potentially affect and change the gene expression profile of key tissues such as skeletal muscle or fat compartments, with relevant implications for their commercial transformation and consumption [1]. These alterations, due to nutritional interventions, may rewire multiple regulatory networks in nutrient metabolism, thus affecting messenger RNA (mRNA) transcription, splicing, trafficking and further synthesis of derived proteins [2,3]. Gene expression regulation can also be mediated through post-transcriptional regulation, of which microRNAs (miRNAs) are key effectors. miRNAs are small non-coding RNAs of ~22 nucleotides long that are able to bind to specific sequences of the 3’ untranslated regions (3’ UTRs) of targeted mRNAs and trigger their degradation and/or inhibit their translation [4]. Thousands of miRNAs can be found on online databases such as *miRBase*, a searchable collection of published miRNA sequences and their annotation. As of its release v22.1, there are 38,589 miRNA entries published over 271 species, including 408 precursors and 457 mature miRNAs for *Sus scrofa* (pig) [5]. Besides, the development of next generation sequencing (NGS) technologies has provided a better understanding of the genome organization, structure, function, and evolution in livestock animals. Nowadays, it is commonly used to study complex traits to improve livestock production efficiency and reproductive health [6].

Pigs are one of the most important agricultural livestock animals for meat production, accounting to a total of 122.5 million tons globally in 2021. Food and Agriculture Organization (FAO) also highlighted that the recent expansion of world meat output was mainly driven by the increase in pork output [7]. Porcine fatness or leanness are considered as relevant target traits for selection since they could impact productive and reproductive performance, as well as meat quality [8]. Specific porcine breeds, such as Landrace, have been extensively selected to increase lean meat production and reduce fat deposition [9]. Although this may improve overall pig production efficiency, such breeding programs may negatively affect meat quality traits like juiciness, tenderness, flavor and overall sensory quality of pork [10]. For a more efficient production, fats and oils are supplemented on diets, as they contain 2.25 times more energy than cereal grains, which further increases energy density and reduces feed intake [11]. Additionally, the fatty acid content of the carcass is directly influenced by the dietary fats ingested by pigs, mimicking the fatty acid composition of the diet [12]. However, there is still limited knowledge on how mRNAs and miRNAs interact to regulate fatty acid metabolism pathways, or how diet and fatty acid content might influence their expression profiles, especially on Iberian pigs [13]. Moreover, as the excessive supplementation of omega-6 (n-6) PUFAs becomes more prevalent on commercial pig diets, this pro-inflammatory PUFAs can impose risk of developing inflammatory diseases such as cardiovascular diseases, diabetes or obesity [14,15]. Regulation of n-6 PUFA-derived metabolites can be done through balancing the ratio between these and omega-3 (n-3) PUFAs, which counteract pro-inflammatory responses elicited by the excess of n-6 PUFAs [16].

In order to better understand putative regulatory relationships between mRNAs and miRNAs related to changes in the n-6/n-3 PUFAs muscle composition in pigs, we identified differentially expressed (DE) mRNAs and miRNAs in skeletal muscle tissue from a population of Iberian × Duroc pigs with high and low values of n-6/n-3 PUFAs ratio. Additionally, we characterized putative mRNA-miRNA transcriptomic interactions using computational prediction and regulatory network analyses.

## Materials and methods

### Animal material

A total of 20 *longissimus dorsi* (LD) skeletal muscle samples were obtained from an experimental backcross population of Iberian and Duroc pigs, as previously described by Martinez-Montes *et al*. [17]. Pigs were housed following standard intensive system according to European directives on animal welfare, and were fed *ad libitum* with a cereal-based commercial diet. Muscle samples were collected immediately after slaughter, snap-frozen in liquid nitrogen and stored at -80ºC until further use. Fatty acids profiling was performed by using gas chromatography of methyl esters protocol on 200 g of LD muscle. Sampled animals were selected based on their analyzed values for n-6/n-3 PUFAs ratio and a total of 10 with highest (H) and 10 with lowest (L) n-6/n-3 ratio values were kept for further analyses [18]. A similar number of males and females were present in each group (S1 Table) and the use of siblings within each group was avoided. A summary of the measured phenotypes in the selected animals is available at S1 Table.

#### Ethics statement

All animal procedures were performed according to the Spanish Policy for Animal Protection RD1201/05, which meets the European Union Directive 86/609 about the protection of animals used in experimentation. The protocol was approved by the Committee on the Ethics of Animal Experiments of the Instituto Nacional de Investigación y Tecnología Agraria y Alimentaria CEEA (Permit Number: 2014/026).

### RNA isolation, library preparation, and sequencing of total and small RNAs

#### Total RNA

The LD skeletal muscle samples were submerged in liquid nitrogen, pulverized using a mortar and pestle, and subsequently homogenized in 1 ml of TRI Reagent (Thermo Fisher Scientific, Barcelona, Spain). The RiboPure kit (Ambion, Austin, Texas, USA) was used to isolate the total RNA fraction, and its concentration and purity were determined with a Nanodrop ND-1000 spectrophotometer (Thermo Fisher Scientific, Barcelona, Spain). RNA integrity was assessed with a Bioanalyzer-2100 equipment (Agilent Technologies Inc., Santa Clara, California, USA), using the Agilent RNA 6000 Nano Kit (Agilent Technologies, Inc., Santa Clara, California, USA). Libraries were prepared with the TruSeq SBS Kit v3-HS (Illumina Inc., California, USA) and a minimum of 30 million hits of 75 bp-length paired-end reads were acquired per sample using an Illumina HiSeq 3000/4000 equipment (CNAG-CRG, Barcelona, Centro Nacional de Análisis Genómico; https://www.cnag.crg.eu).

#### Small RNA

The extraction of total RNA, including miRNA and small RNA, was performed with the same muscle tissue material employed for total RNA sequencing and using the miRNeasy Kit (QIAGEN, Germantown, Maryland, USA) following manufacturer’s specifications. More specifically, approximately 50 mg of tissue per sample was disrupted and homogenized in 700 μl of QIAzol Lysis Reagent (QIAGEN, Germantown, Maryland, USA) using 2ml Lysing matrix D tubes (MP Biomedicals, Santa Ana, CA) and a Precellys 24 instrument (Bertin Technologies, Rockville, MD). After RNA isolation following the miRNeasy protocol, the extracted RNA molecules were eluted in 30 μl of water. The concentration, purity, and RNA integrity were assessed as per aforementioned for total RNAs. A minimum of 10 million hits of 50 bp-length single-end read were acquired per sample using the same sequencing equipment used for mRNA libraries.

### RNA-Seq and miRNA-Seq data processing

Raw mRNA and miRNA sequences were subjected to quality control through the FastQC tool [19]. In order to remove the Illumina adapters used during library preparation and sequencing, reads were trimmed using the Cutadapt software v0.9.5 [20]. RNA-Seq data sequence alignment was performed against the reference pig genome (*Sscrofa11*.*1*) by using the STAR [21] aligner with default parameters. Sequences were then quantified with the RSEM software [22]. On the other hand, for miRNA-Seq data, sequence alignment was performed against the reference pig genome (*Sscrofa11*.*1* and miRBase 22.1) by using Bowtie^23^ aligner and the following specifications for aligning short miRNA reads were taken into consideration: 1) allowing no mismatches in the alignment, 2) removing reads with more than 20 putative mapping sites, and 3) reporting first single best stratum alignment (bowtie -n 0 -l 25 -m 20 -k 1—best—strata) [23,24]. Quantification of aligned miRNA reads were performed using HTSEQ software [25]. Only mRNAs and miRNAs with an overall expression across all samples higher than 20 counts were considered for subsequent differential expression analyses [26].

Differential gene expression analyses between the H and L groups from both RNA-Seq and miRNA-Seq data were performed with the DESeq2 software [26], including sex and batch effects as covariates in the linear model (S1 Table). Both mRNAs and miRNAs from differential expression analyses were considered significant at an absolute fold-change (FC) > 1.5 and adjusted *p*-value < 0.05. We considered the H group as reference, meaning that any gene upregulation would imply its overexpression in the L group, resulting in a positive fold change, and vice versa.

### Gene ontology and pathway enrichment analysis

Differentially expressed mRNA genes analyzed between H and L groups were subjected to Gene ontology (GO) and pathway enrichment analyses using Cytoscape v3.7.1 software with the ClueGO v.2.5.4 plug-in application to determine enriched Biological Process terms [27,28]. Identification of enriched terms was done using a one-sided hypergeometric test of significance, with a false discovery rate approach for multiple testing correction [29].

### Co-expression network analysis between mRNAs and miRNAs

A co-expression network between mRNA and miRNA expression profiles was built according to the established pipeline as previously reported by Mármol-Sánchez *et al*. [24]. The Partial Correlation with Information Theory (PCIT) network inference algorithm was used to recognize meaningful gene-to-gene interactions by employing first-order partial correlation coefficients obtained for each trio of genes in conjunction with an information theory technique [30,31]. To do so, we calculated the Pearson pairwise correlation coefficients (*r*) for each expressed miRNA and DE mRNA between H and L groups. Assuming that miRNAs can biologically suppress mRNA expression, we reported only those co-expressed miRNA-to-mRNA pairs showing an *r* value < -0.50. To further retain only relevant miRNA-to-mRNA correlations with biological meaning, the seed of the annotated porcine mature miRNAs (7mer-m8, from 2^nd^ to 8^th^ 5’ nucleotides) were reverse-complemented and interrogated along the annotated 3’ UTRs (Sscrofa 11.1; http://www.ensembl.org./biomart) of porcine mRNA genes, by making use of the SeqKit toolkit [32]. We also investigated whether the mRNAs predicted to interact with miRNAs showed meaningful expression correlations with other mRNA-encoding genes. We only kept the mRNA pairs with |*r|* > 0.7 as determined by the PCIT algorithm. The more stringent threshold imposed for mRNA-to-mRNA expression associations as compared to miRNA-to-mRNA predicted interactions (*r* < -0.50) was motivated by the fact that the expression correlation between mRNA pairs is commonly of great magnitude than that of miRNA-to-mRNA interactions [33].

## Results

### Differentially expressed genes and miRNAs

**Fig 1:**
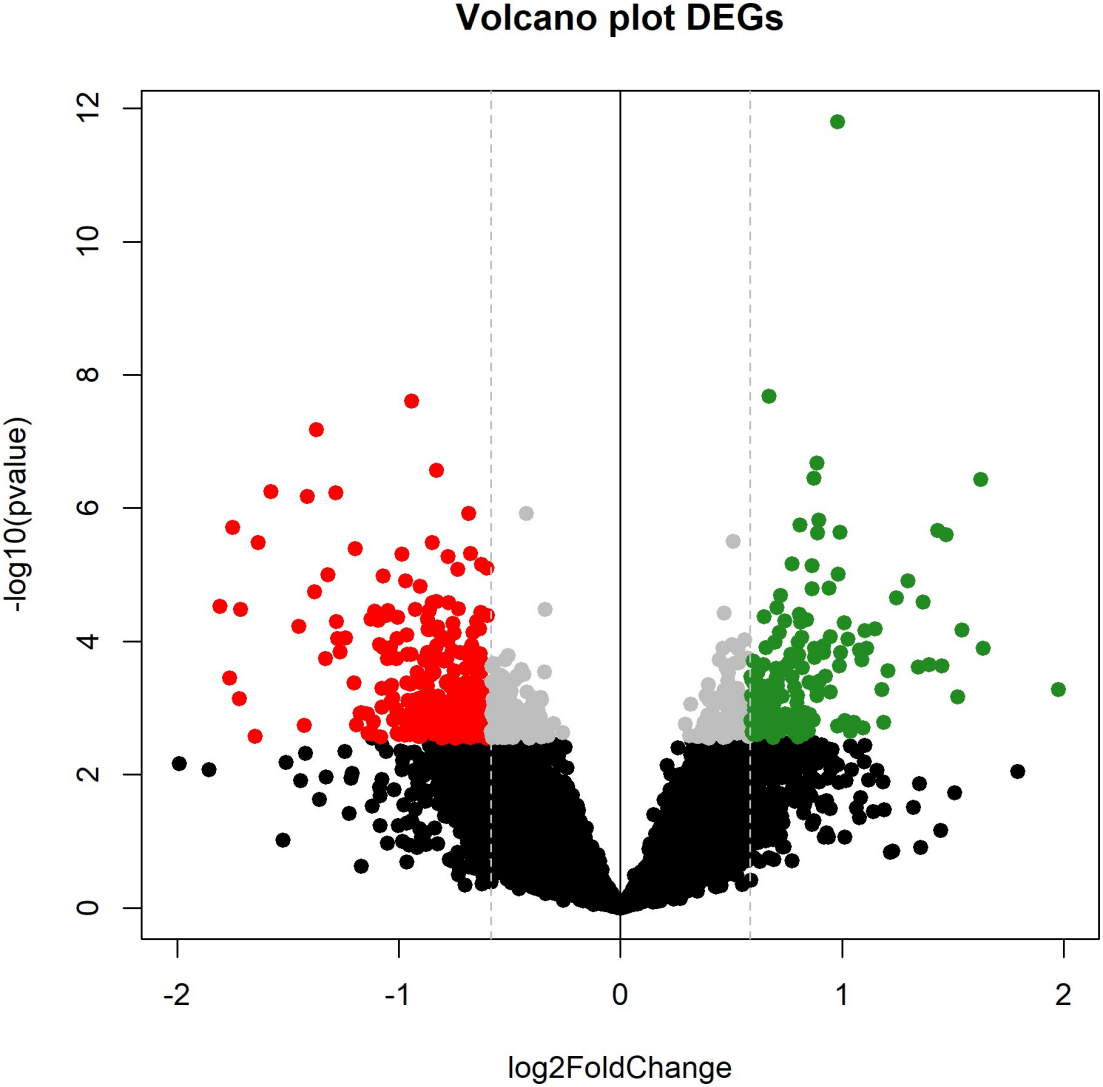
Volcano plot showing differentially expressed mRNA genes (DEGs) with an absolute fold change > 1.5 and adjusted *p*-value < 0.05 after comparing pigs with High (H) and Low (L) profiles of n-6/n-3 PUFAs ratio in *longissimus dorsi* skeletal muscle. Upregulated genes (in green) correspond to genes overexpressed in L pigs, and vice versa.

Out of 11,521 porcine mRNAs detected as sufficiently expressed, a total of 432 differentially expressed genes (DEGs) were obtained between H and L pigs according to their n-6/n-3 PUFA ratio (S2 Table), with 157 and 275 mRNAs being upregulated and downregulated in L pigs with respect to H pigs, respectively (Fig 1). From the 457 annotated porcine miRNAs, a |FC| > 1.5 threshold for changes in expression between H and L pigs showed no DE miRNAs for the PUFA ratio trait. When a less stringent FC threshold was considered (|FC| > 1.2), 4 DE miRNAs were identified: *ssc-miR-15b*, *ssc-mir30a-3p*, *ssc-miR-30e-3p* and *ssc-miR-7142-3p* (S3 Table).

### Functional analysis and pathway enrichment of DEGs

**Fig 2:**
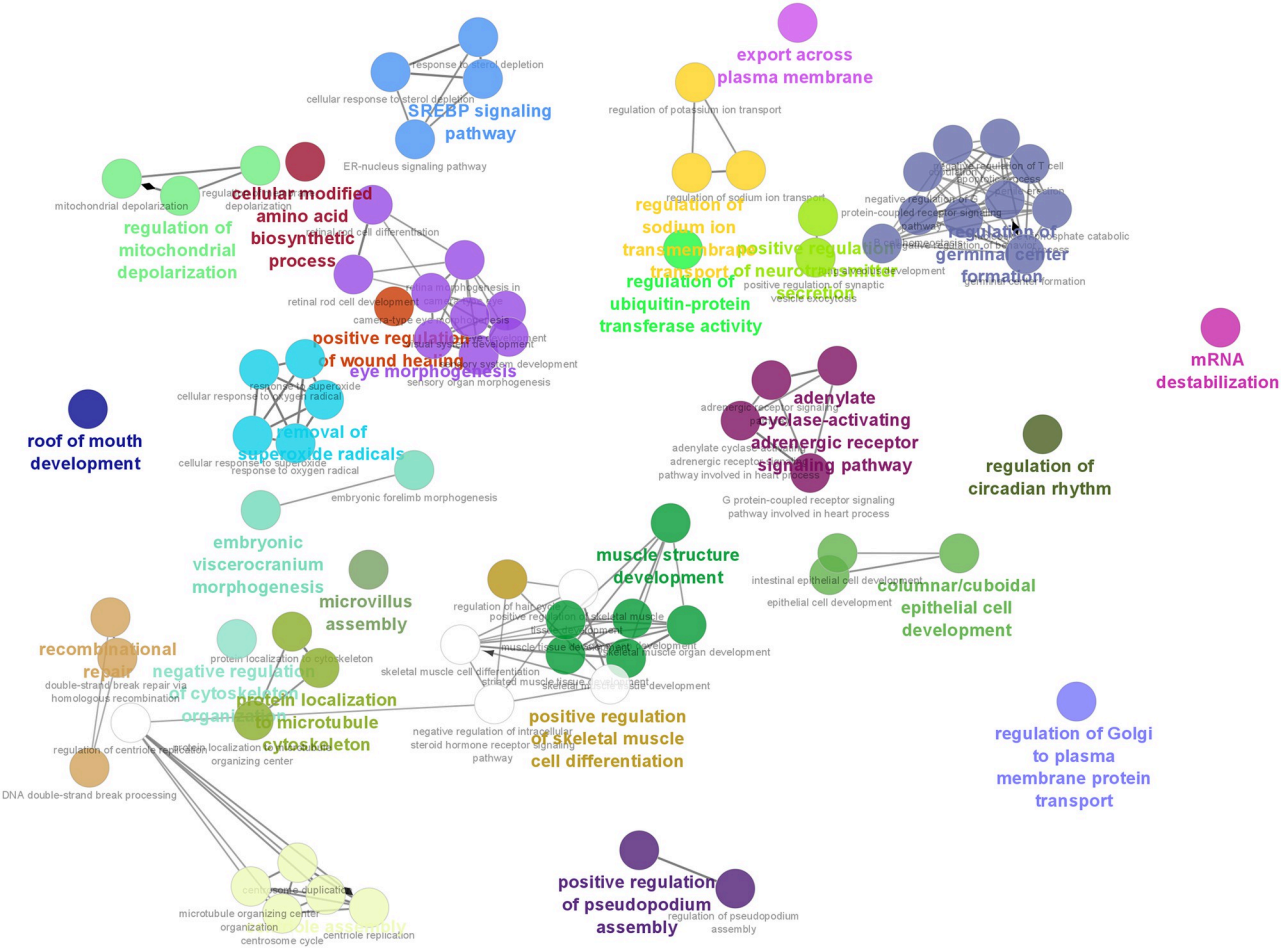
Enriched gene ontologies using DEGs after comparing RNA-Seq gene expression profiles of H and L pigs and their related biological processes. Significant unique GO terms (adjusted *p*-value < 0.05) are obtained from the ClueGO plug-in embedded on the Cytoscape software.

A total of 80 significant unique GO terms (adjusted *p*-value < 0.05) were detected for DEGs related to H and L pigs for the n-6/n-3 PUFA ratio trait. A full list of enriched GO terms is shown in S4 Table. The significant biological processes highlighted were related to muscle structure development (*GO*:*0061061*), positive regulation of skeletal muscle cell differentiation (*GO*:*2001016*), SREBP signaling pathway (*GO*:*0032933*) and adrenergic receptor signaling pathway (*GO*:*0071875*), among others, as shown in Fig 2.

### mRNA-miRNA co-expression regulatory network

A total of 196 miRNAs were detected as significantly co-expressed (*r* < -0.50) with the differentially expressed mRNA genes for n-6/n-3 PUFAs ratio (S5 Table). Among the 196 detected miRNAs, we focused on the 4 DE miRNAs previously highlighted (see Results above, S3 Table) and used them for predicting binding sites of their mature miRNA seeds to the 3’ UTR region of putative DE mRNA targets (S6–S8 Tables). Almost half (214 out of 432, 49.54%) of the DEGs showed putative binding sites in their 3’ UTRs for the seed region of the 4 DE miRNAs (Table 1 and S3 Table).

**Table 1:** Number of putative targeted DE mRNAs (DEGS) with predicted binding sites for DE miRNAs between pigs with High (H) and Low (L) n-6/n-3 PUFAs ratio in *longissimus dorsi* skeletal muscle.

DE miRNAs	Number of targeted DEGs^a^	% over total DEGs
***ssc-mir-15b***	125	28.94%
***ssc-miR-30a-3p***	130	30.09%
***ssc-miR-30e-3p***		
***ssc-miR-7142-3p***	54	12.50%

^a^Differentially expressed genes (DEGs) = 432 in total; The *ssc-miR-30a-3p* and *ssc-miR-30e-3p* have the same mature miRNA seed (7mer-m8, from 2^nd^ to 8^th^ 5’ nucleotides).

Further combining relevant miRNA-mRNA expression correlations according to the PCIT algorithm (*r* < -0.50) and 3’ UTR region seed matching, 2 out of the 4 DE miRNAs showed meaningful co-expression with two DEGs: *ssc-miR-15b* was predicted to bind to the 3’ UTR of the arresting domain containing 3 (*ARRDC3*) gene, while *ssc-miR-7142-3p* was predicted to bind the 3’ UTR of the methyltransferase-like 21C (*METTL21C*) gene (S9 Table).

**Fig 3:**
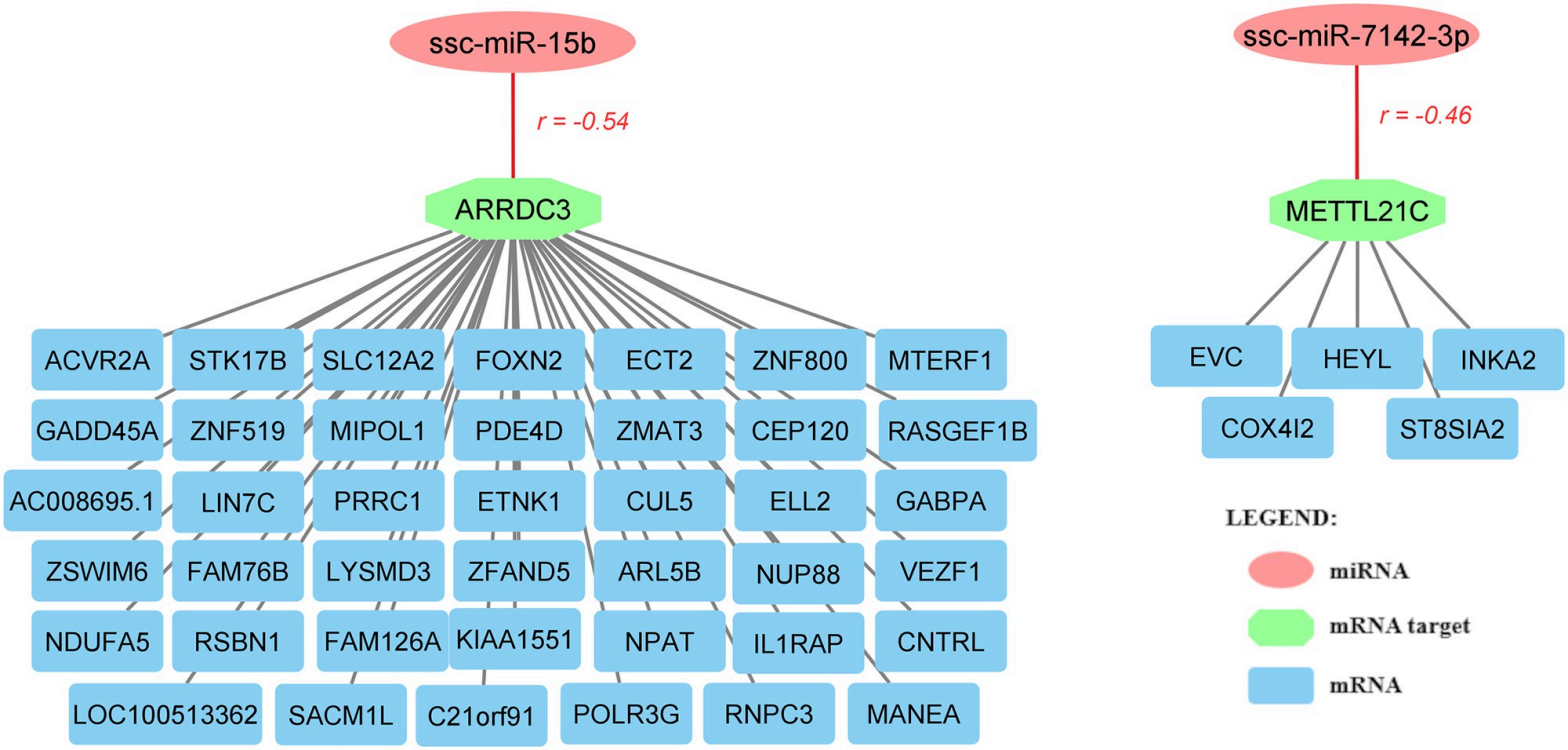
Meaningful co-expression network between miRNA-to-mRNA and mRNA-to-mRNA genes. *r* = Pearson correlation value; lines in red for miRNA-to-mRNA interactions denote a negative correlation.

Several other genes were also significantly associated with the expression of these two DEGs (*ARRDC3* and *METL21C*, S10 and S11 Tables). As shown in Fig 3, the *ARRDC3* gene showed meaningful correlation with 41 differentially expressed mRNAs (S10 Table), whereas the *METTL21C* gene was significantly correlated with 5 DEGs (S11 Table). The related functions and associations to lipid metabolism, immunity, and/or inflammation of these DEGs aresummarized in S12 Table.

## Discussion

### DEGs and their relationship to lipid-mediated expression and immunomodulation

Our results after GO enrichment analyses of DEGs (S4 Table) showed GO terms mostly related to muscle growth and differentiation, glucose and lipid metabolism. Some of the genes related to muscle tissue and structure development pathways were also reported in human, mice and ruminants (i.e. cattle, sheep, lamb). The aryl hydrocarbon receptor nuclear translocator like (*ARNTL*), a gene that regulates the circadian release of PUFAs and modulates feeding behavior in mice, alongside with forkhead box N2 (*FOXN2*), are associated with obesity [34,35]. Another interesting gene was the diaphanous related formin 1 (*DIAPH1*), which is regulated upon nutritional intervention with long chain PUFAs (n-6 and n-3) and it is reported to be involved in lipid metabolism in cattle [36]. Supplementation of flaxseed or fish oil can increase the expression of the guanidinoacetate N-methyltransferase (*GAMT*) gene, which is involved in folate-homocysteine metabolism in embryos and liver of pregnant mice [37]. The absence of n-3 PUFA in rodents (i.e. DHA) has been reported to affect cognitive brain function and a few of its synaptomes including homer scaffold protein 1 (*HOMER1*) [38]. The Kruppel like factor 5 (*KLF5*) gene regulates muscle differentiation in myoblasts and controls lipid metabolism in mature skeletal muscle in mice [39,40]. In addition, GWAS analyses in human metabolic syndrome discovered the association of the strawberry notch homolog 1 (*SBNO1*) gene on plasma high-density lipoprotein cholesterol concentration, whereas the vestigial like family member 2 (*VGLL2*) gene was linked to the fatty acids profile in sheep [41,42]. The T-box transcription factor 1 (*TBX1*) gene, together with *miR-193a-3p*/*TGF-β2*, was found to drive iron-dependent cell death ferroptosis through the accumulation of lipid peroxides in neonates [43,44]. Abundancy on n-3 PUFA in cattle is reported to increase the gene expression of insulin-like growth factors such as the insulin-like growth factor binding protein 5 (*IGFBP-5*) and further influence reproductive performance [45]. Other genes like the glycerophosphocholine phosphodiesterase 1 (*GPCPD1*), 3-hydroxy-3-methylglutaryl-CoA reductase (*HMGCR*), or phosphoglucomutase 5 (*PGM5*), are related to glycophospholipid formation, cholesterol synthesis and glycolysis [46–49].

Another relevant GO term that can be highlighted from our results is the SREBP signaling pathway. Sterol regulatory-element binding proteins (SREBPs) are transcription factors that regulate the expression profiles of genes that are involved in lipid synthesis, energy storage and cholesterol regulation. When these proteins are activated, they can trigger lipid-mediated cellular stress that can cause metabolic diseases such as obesity, atherosclerosis, diabetes mellitus, inflammation, and organ fibrosis [50,51].

On the other hand, our enrichment analyses also emphasized the adenylate cyclase-activating adrenergic receptor signaling pathway based on the DEGs involved. Adrenergic receptors play a vital role in mediating stress-induced signals, in immunomodulation and in stress-related behavioral changes [52,53]. This pathway also triggers the formation of cyclic-adenosine 3′,5′-monophosphate (cAMP), which regulates intracellular metabolism and it is linked to glycolysis [54,55]. Stimulation of both SREBP signaling pathway and adrenergic receptor signaling pathway could also be related to the pro-inflammatory role of n-6 PUFA. Addition of n-6 PUFA increased the β-adrenergic receptor binding and adenylate cyclase activity in pig adipocyte plasma membrane [56]. Furthermore, it was also reported that over supplementation of n-6 PUFAs in swine diets can stimulate the innate immune response and acute inflammatory response [57].

### Association of differentially expressed porcine miRNAs to adipogenesis and inflammation

We obtained a total of 4 DE miRNAs (*ssc-miR-30a-3p*, *ssc-miR-30e-3p*, *ssc-miR-15b* and *ssc-miR-7142-3p*) between high and low n-6/n-3 PUFA ratio contrast on porcine skeletal muscle. The expression of *miR-30a* in pigs has been associated to adipocyte formation, fat deposition, myogenic differentiation and immune system [58–62]. *miR-30a* may also be related to cellular response to infection, immune modulation and pathological processes since it was detected on multiple pig-related viral studies concerning porcine parvovirus, porcine reproductive and respiratory syndrome virus or H1N1 swine influenza A virus [63–65]. A study on a minipig obesity model also demonstrated how *miR-30a* could regulate the expression of genes related to adipogenesis and low-grade chronic inflammation in obesity [58,66,67]. This was in accordance to a previous report by our team, in which we predicted that *miR-30a* could potentially bind to and regulate the mRNA of porcine *ELOVL* fatty acid elongase 6 (*ELOVL6*) gene, which is responsible of the elongation of PUFAs and *de novo* lipogenesis [68]. However, the *ELOVL6* gene was not among the detected DEGs in the current study (S2 Table), thus suggesting that differences in *miR-30a* expression are not affecting the *ELOVL6* mRNA levels, but might inhibit its translation. As a member of *miR-30* family, *ssc-miR-30e* also targets mRNA genes that are related to skeletal muscle growth, energy metabolism and increased feed efficiency in swine [69,70]. A few reports on pigs have also elucidated the role of *miR-30e* on binding to mRNA transcripts from genes related to pathogenesis, virus-host interactions and immune response [71–73].

On the other hand, *mir-15b* is mainly associated to blood vessel formation (angiogenesis), tumor growth and cellular ATP level modulation. Metabolites obtained from n-6 PUFAs could promote angiogenesis by increasing expression of transcription growth factors (i.e. *TGF-β*), whereas n-3-PUFA-derived substances contain anti-angiogenic, anti-inflammatory and antitumor properties [74–76]. Besides, *ssc-mir-7142-3p* is a mirtron located in the intronic fraction of the microtubule affinity regulating kinase 2 (*MARK2*) gene. This miRNA has been detected in lung tissue infected with *Actinobacillus pleuropneumoniae* and its differential expression has been associated to the overexpression of the retinol binding protein 4 (*RBP4*) gene [77,78]. *RBP4*, mainly secreted by the liver and adipocytes, is a transporter of vitamin A and it is involved in various pathophysiological processes, such as obesity, insulin resistance and cardiovascular diseases, demonstrating a strong association of this mirtron to inflammatory-related processes [78].

### Meaningful miRNA-to-mRNA regulatory networks affected by changes in n-6/n-3 ratio

Co-expression network analyses between DE miRNAs and DEGs highlighted 2 miRNAs that could potentially bind to and inhibit the expression of 2 DE mRNAs when comparing pigs with high and low n-6/n-3 PUFA ratio in skeletal muscle. The upregulated DE miRNA *ssc-miR-15b* was predicted to bind to the 3’ UTR of the arrestin domain containing 3 (*ARRDC3*) gene. Arrestins are a small family of multi-faceted protein trafficking adaptors that bind to membrane proteins, which regulate signal transduction at G protein-coupled receptors (GCPR) and promote endocytosis. *ARRDC3* is a known α-arrestin and its activation could be due to nutrient excess or cellular stressors [79,80]. Our results showed that this gene was involved in a few biological processes such as adrenergic receptor signaling pathway, negative regulation of G protein-coupled receptor signaling pathway, negative regulation of behavior and regulation of ubiquitin-protein transferase activity. *ARRDC3* was reported to co-immunoprecipitate and interact with β_2_-adrenergic receptors and facilitate its ubiquitination and degradation [81–83]. In addition, this gene is also involved in obesity development, insulin resistance, body mass regulation, glucose metabolism, adiposity and energy expenditure [84–86].

Meanwhile, the mirtron *ssc-miR-7142-3p* might target the methyltransferase-like 21c (*METTL21C)* mRNA transcripts, which encode for a protein-lysine methyltransferase involved in regulation of myogenesis, muscle function and protein catabolism [87,88]. From our results, there is a strong positive association between high n-3 PUFA concentration and *METTL21C* expression. A decreased expression of this gene was also reported after long-term exercise, in which elevated levels of inflammatory cytokines, oxidative stress, and leukocytosis could be observed [89,90]. From our results, we might hypothesize that the upregulation of *ssc-miR-15b* and downregulation of *ssc-miR-1472-3p*, together with *ARRDC3* downregulation and *METTL21C* upregulation could be linked to low n-6/n-3 PUFA ratio and the production of anti-inflammatory metabolites, stimulating receptors related to stress and immunity. However, further validation among these predicted regulatory networks should be done in order to verify their biological importance in terms of porcine growth and immune response.

### Putative mRNA-to-mRNA correlations highlight genes related to immunity and metabolic stress

Potential correlation and interaction between the two possible target genes of DE miRNAs, *ARRDC3* and *METTL21C*, and DEGs were further investigated. The phosphodiesterase 4D (*PDE4D*), a gene that is associated with the regulation of interleukin production and cAMP-mediated signaling, belongs to the same adrenergic receptor signaling pathway as *ARRDC3* (S4 Table). The tumor inhibition properties of *ARRDC3* are presumably facilitated by linking target substrates such as β-adrenergic receptor and integrin β4 to E3 ligase, in which these target substrates become ubiquintinated and degraded by the proteasome [91].

Both the Hes related family bHLH transcription factor with YRPW motif like (*HEYL*) and EvC ciliary complex subunit 1 (*EVC*) genes were correlated with *METTL21C*, and associated with muscle organ and structure development (S4 Table). One study looked into the changes of gene expression on some signaling pathways that could be affected by the specific knockdown of *METTL21C*, including *HEYL*-targeted Notch pathway. Although it did not affect the expression of *HEYL* gene, they reported that *METTL21C* is a critical component for bone and muscle homeostasis [92]. Our results also showed that both *EVC* and *METTL21C* were upregulated in L pigs. In contrast, an upregulation *EVC* and downregulation *METT21C*, among other differentially expressed genes, were observed in *BRAF*-mutant cell lines, in response to metabolic stress through glucose withdrawal [93].

## Conclusion

The high and low values of n-6/n-3 PUFA ratio on porcine skeletal muscle influence the expression profiles, related biological pathways and transcriptomic correlations and interactions between differentially expressed mRNAs and miRNAs. Predicted co-expression regulatory networks among mRNAs and miRNAs may be attributed to the pro- and anti-inflammatory functions of n-6 and n-3 PUFAs, respectively. Our findings highlighted mRNA genes, miRNAs and enriched pathways that were related to lipids metabolism, cell growth and inflammation, according to differences in muscle n-6/n-3 PUFA ratio.

## Supporting information

S1 Table Phenotypic values of n-6/n-3 PUFA ratio, sex classification, and batch grouping recorded in 20 Iberian x Duroc pigs. (XLSX)

S2 Table Differentially expressed genes (DEGs) according to n-6/n-3 PUFA ratio. (XLSX)

S3 Table Differentially expressed miRNAs (DEmiRNAs) according to n-6/n-3 PUFA ratio. (XLSX)

S4 Table List of Gene Ontology (GO) terms related to DEGs according to n-6/n-3 PUFA ratio. (XLSX)

S5 Table Predicted porcine mature miRNAs co-expression with DEGs. (XLSX)

S6 Table *miR-15b* seed binding interrogation on the 3’ UTR of DEGs. (XLSX)

S7 Table *miR-30a* and *miR-30e* seed binding interrogation on the 3’ UTR of DEGs. (XLSX)

S8 Table *miR-7142* seed binding interrogation on the 3’ UTR of DEGs. (XLSX)

S9 Table Meaningful co-expression network between negatively correlated DEGs and DE miRNAs according to n-6/n-3 PUFA ratio. (XLSX)

S10 Table Meaningful mRNA-mRNA co-expression of *ARRDC3* gene and other DEGs according to n-6/n-3 PUFA ratio. (XLSX)

S11 Table Meaningful mRNA-mRNA co-expression of *METTL21C* gene and other DEGs according to n-6/n-3 PUFA ratio. (XLSX)

S12 Table List of genes significantly correlated with *ARRDC3* and *METTL21C* and their related functions. (XLSX)
